# Dissociating functional brain networks by decoding the between-subject variability

**DOI:** 10.1016/j.neuroimage.2008.12.017

**Published:** 2009-04-01

**Authors:** Mohamed L. Seghier, Cathy J. Price

**Affiliations:** Wellcome Trust Centre for Neuroimaging, Wellcome Department of Imaging Neuroscience, Institute of Neurology, UCL, 12 Queen Square, London WC1N 3BG, UK

**Keywords:** Functional MRI, Fuzzy clustering, Between-subject variance, Inter-individual variability, Across-subject variability, Semantic decision, Language, Networks, Default network, Second-level analysis, Cognitive subtractions

## Abstract

In this study we illustrate how the functional networks involved in a single task (e.g. the sensory, cognitive and motor components) can be segregated without cognitive subtractions at the second-level. The method used is based on meaningful variability in the patterns of activation between subjects with the assumption that regions belonging to the same network will have comparable variations from subject to subject. fMRI data were collected from thirty nine healthy volunteers who were asked to indicate with a button press if visually presented words were semantically related or not. Voxels were classified according to the similarity in their patterns of between-subject variance using a second-level unsupervised fuzzy clustering algorithm. The results were compared to those identified by cognitive subtractions of multiple conditions tested in the same set of subjects. This illustrated that the second-level clustering approach (on activation for a single task) was able to identify the functional networks observed using cognitive subtractions (e.g. those associated with vision, semantic associations or motor processing). In addition the fuzzy clustering approach revealed other networks that were not dissociated by the cognitive subtraction approach (e.g. those associated with high- and low-level visual processing and oculomotor movements). We discuss the potential applications of our method which include the identification of “hidden” or unpredicted networks as well as the identification of systems level signatures for different subgroupings of clinical and healthy populations.

## Introduction

Functional magnetic resonance imaging studies are based on the premise that different types of cognitive and sensorimotor processes are supported by functionally segregated networks that integrate in unique combinations to allow the processing of an infinite number of tasks. For example, a task that entails categorisation of visual stimuli with a finger-press response involves a minimum of three functional networks that support (i) visual processing (ii) categorisation and (iii) the finger press. Typically, these networks are segregated following the well-known logic of “cognitive subtraction” that compares neuronal activation during a range of different conditions, each tapping a different process (e.g. visual processing without a manual response or manual response without visual processing). The problems associated with cognitive subtractions, which assume that brain functions are additive, are well recognised (for a critical review see Friston et al., 1996; Newman et al., 2001; Sartori and Umilta, 2000; Sidtis et al., 1999). The limitation we address here is that cognitive subtraction in fMRI only targets networks that are anticipated *a priori* (i.e. those explicitly considered during condition selection), thereby leaving hidden other potentially interesting networks that are not predicted on the basis of *a priori* knowledge. To address this limitation, we propose a novel approach that segregates functional networks on the basis of between-subject variability in one representative measure of brain activity for a single task/condition, without the need for other conditions or cognitive subtractions. In other words, we argue here that segregating networks for a given task/condition can be achieved by considering the between-subject dimension “only” without any *a priori* knowledge.

**Fig. 1:**
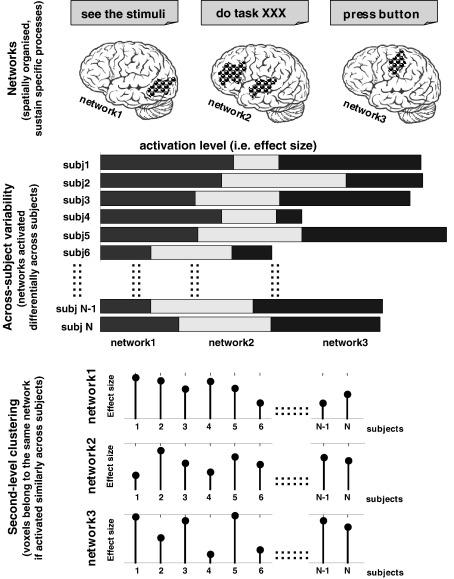
A schematic view of a hypothetical fMRI experiment with N subjects (see Introduction section for more details).

To illustrate our rationale, we start by considering the impact of between-subject variability on our hypothetical visual categorisation task (Fig. 1). In this task, we assume that there are three integrated functional networks. Network 1 involves visual and perceptual processing; Network 2 involves categorisation and decision making; and Network 3 involves finger press responses. We further assume that all subjects engage the three functional networks “differently” when performing the task, but, because the networks are functionally segregated (and spatially distinct), the level of activation in one network is not completely correlated with the level of activation in the other networks (i.e. the networks operate independently). Therefore a single subject may have high activation relative to the other subjects in one network but low activation relative to the other subjects in another network. For example, subject 4 in Fig. 1 has relatively high activation for visual processing but relatively low activation for motor processing (see horizontal bars in Fig. 1). This results from individual differences in how a subject performs the same task, as commonly observed in multi-subject fMRI studies (for more details see Kherif et al., 2003; Miller and Van Horn, 2007; Nadeau et al., 1998; Seghier et al., 2007, 2008a).

There are two critical points to note. First, activation at any given voxel will have a between-subject variability profile (BSVP) which reflects the range of activation across subjects (see vertical bars in Fig. 1). Second, if this voxel is part of a functional network, then we expect that other voxels in the same network will have a similar BSVP. In contrast, we expect that when two voxels are parts of different functional networks, they will have different BSVPs. The segregation of different functional networks then reduces to the output from clustering algorithms that cluster/aggregate voxels with similar between-subject variability profiles. In other words, the rationale behind our work can be rephrased as follows: there is a meaningful structure in the between-subject variability that can be decoded by assuming that regions belonging to the same network will have comparable variations (i.e. BSVPs) from subject to subject.

The key assumption in the above rationale is that the between-subject variability profile (BSVP) is more dominated by the engagement of a particular functional network than by other inevitable sources of variability from, for example, differences in local anatomy or regional hemodynamics. Support for this assumption comes from previous studies that have used between-subject variability to identify large-scale cortical networks during rest (Damoiseaux et al., 2006), object recognition (Sugiura et al., 2007), word and symbol perception (Reinke et al., 2008), overt word reading (Seghier et al., 2008b), emotional memory suppression (Depue et al., 2007), and brain structure (Mechelli et al., 2005). Our approach is based on the same assumptions as these studies (c.f. Seghier et al., 2008b) but our perspective is fundamentally different: we want to dissociate, in the between-subject dimension, sets of functional networks that combine together to support a given task. Our approach also contrasts with previous studies that explored the between-subject variability in the time domain (e.g. Hasson et al., 2004; Hejnar et al., 2007; Jaaskelainen et al., 2008; Pinel et al., 2007) because we define each subject's contribution by one representative measure only (e.g. effect size or percent signal change for the one task of interest), hence eliminating the time dimension (i.e. the within-subject variability) and focusing only on the between-subject dimension (as illustrated in Fig. 1). Our focus on between-subject variability is also motivated by the fact that, in fMRI data, between-subject variability generally dominates within-subject variability (e.g. Miller et al., 2002; Smith et al., 2005).

To summarise, we aim to dissociate functional networks, in the absence of any *a priori* knowledge at the second-level, by using a data-driven (clustering) approach to decode the between-subject variability during the execution of a single task. To illustrate our approach we report an fMRI study of visual word categorisation in thirty-nine subjects, and systematically compare the results of the conventional cognitive subtraction approach with our between-subject variability clustering approach (referred to here as second-level clustering). This demonstrates that the second-level clustering identifies all the expected networks, and in addition reveals dissociations that were not predicted *a priori*. Having demonstrated the feasibility of this approach, we describe how it could be applied to the subgrouping of clinical populations, particularly when the use of a single task maximizes sensitivity while minimizing patient time in the scanner.

## Methods

The aim of our experiment was to identify expected and unexpected functional subsystems (networks) that underlie successful performance on a visual categorisation task that is commonly used in clinical practice. The task, known as “*pyramids and palm trees*” (Howard and Patterson, 1992), involves three visually presented words, with one target above and two choices below. The subject is required to indicate which of the two choices below is most closely related to the target above. For example, when the target is “pyramid” and the two alternative choices are “oak tree” or “palm tree”, the correct response is “palm tree” because pyramids are more likely to be found in the vicinity of palm trees than oak trees. This choice of task is motivated by its clinical application in standard behavioural assessments (e.g. Swinburn et al., 2004) and its use as a test of semantic function in many previous functional imaging studies (e.g. Mummery et al., 1998; Vandenberghe et al., 1996; Vandenbulcke et al., 2007).

### Subjects

Thirty-nine healthy right-handed subjects (15 males, 24 females) with a wide age range (30 ± 19 years, age range 13–74 years) gave written informed consent to participate in this study. Age range was deliberately wide for additional sources of between-subject variability (see below for more details). Subjects were native English speakers, had normal or corrected-to-normal vision, with no history of neurological or psychiatric disorders. The study was approved by the National Hospital and Institute of Neurology's joint medical ethics committee.

### Task analysis

**Fig. 2:**
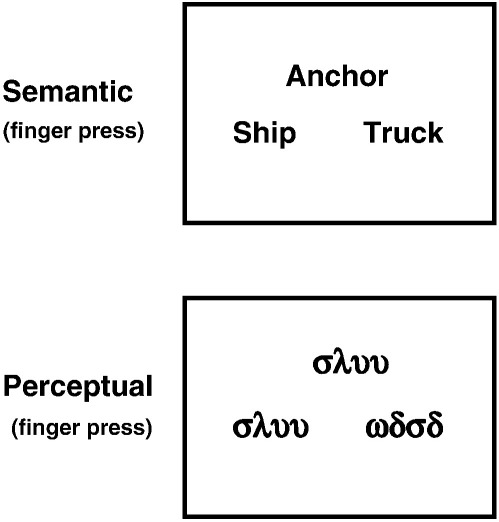
Examples of stimuli used in our paradigm: the activation task (semantic decision on written familiar object names) and the baseline (perceptual matching of unfamiliar Greek symbols).

The pyramids and palm trees task is expected to involve a minimum of (i) visual and perceptual processing; (ii) semantic associations; (iii) categorisation and decision making; (iv) finger press response. Each of these networks may divide into other expected or unexpected functional subcomponents. To generate *a priori* hypotheses for interpreting the results of our clustering algorithm, we start with a conventional cognitive subtraction approach that included one activation condition and one baseline condition (see Fig. 2), interspersed with fixation. The activation condition consisted of matching written object names according to the closest semantic relationship. The baseline consisted of blocks of perceptual matching of unfamiliar Greek letters according to the visual attributes. As in previous studies (e.g. Vandenberghe et al., 1996), it served to control for visuo-attentional and sensorimotor processes involved in semantic categorisation tasks (for a similar rationale see (Cousin et al., 2007; Seghier et al., 2008a, 2004). We also deliberately introduced two between-subject variables to provide an additional test for the clustering algorithm. The first between-subject variable related to the motor response: 27 subjects (12 males, 15 females) were instructed to respond with the index and middle fingers on their right hand and 12 subjects (3 males, 9 females) were instructed to respond with the index and middle fingers on their left hand. This “external” manipulation of the motor output could then be used as a landmark (i.e. witness) of the success of the network segregation. In other words, our second-level clustering should reveal differences in the involvement of the left-hand and right-hand sensorimotor networks. Our second between-subject variable was the age of the participants, this ranged from 13–74 years. Note that the external manipulation (i.e. motor output and age) of the sources of variability is not needed *per se* for the use of the second-level clustering but was included here to validate the success of our method.

### Paradigm and stimuli

Data for the different conditions shown in Fig. 2 were collected in two separate scanning runs/sessions with the order of conditions counterbalanced within and across session. Within each session, there were 24 blocks of stimuli, each lasting 18.8 s with an additional 12 blocks of fixation, each lasting 14.4 s and occurring every two stimulus blocks. Over the experiment, there were 16 blocks presenting written object names and 8 blocks presenting strings of unfamiliar Greek symbols. In addition, there were 24 blocks presenting pictures of objects and non-objects that were not included in the analysis reported here. Each block was preceded by a written instruction (e.g. “match words” which stayed on the screen for 3.6 s). Each stimulus (trial) stayed on the screen for 4.3 s. Hence each 18.8 s block was 3.6 s of instruction and 15.2 s of stimuli. The participants were asked to indicate whether (i) the stimulus on the lower-left or lower-right was more semantically related to the stimulus above (e.g. is “truck” or “ship” most closely related to “anchor”) or (ii) the unfamiliar symbols on the lower-left or lower-right were visually identical to the ones above. Responses were recorded using a button box held under either the right or left hand (see above) with a left finger press indicating the lower left stimulus and a right finger press indicating the lower right stimulus. To ensure that the task was understood correctly, all subjects were provided with detailed instructions and underwent a short training session before entering the scanner with a different set of stimuli.

### MRI acquisition

Data were acquired on a 1.5 T Siemens system (Siemens Medical Systems, Erlangen, Germany). Functional imaging consisted of an EPI GRE sequence (TR = 3600 ms, TE = 50 ms, Flip = 90°, FOV = 192 mm, matrix = 64 × 64, 40 axial slices with 3 × 3 × 3 mm^3^ voxel size). The acquired multi-slice volume was positioned on sagittal scout images. Functional scanning was always preceded by 14.4 s of dummy scans to ensure tissue steady-state magnetization. After functional data collection, anatomical acquisition was obtained to cover the whole brain at high resolution using a T1-weighted sequence to acquire 176 slices with a voxel size of 1 × 1 × 1 mm^3^.

### Data (first-level) analysis

Data processing and statistical analyses were carried out with the Statistical Parametric Mapping SPM5 software package (Wellcome Trust Centre for Neuroimaging, London UK, http://www.fil.ion.ucl.ac.uk/spm/). All functional volumes were spatially realigned, un-warped, normalised to the MNI space, and smoothed with an isotropic 6-mm FWHM Gaussian kernel, with a resulting voxel size of 2 × 2 × 2 mm^3^. The unified normalisation–segmentation procedure of SPM5 was used during the normalisation of the functional brains (Ashburner and Friston, 2005). Time-series from each voxel were high-pass filtered (1/128 Hz cut-off) to remove low-frequency noise and signal drift. The pre-processed functional volumes for each subject were then submitted to a fixed-effects analysis, using the general linear model at each voxel. Each stimulus onset (except fixation) was modelled as an event encoded in condition-specific ‘Dirac Delta-Functions’. The resulting stimulus functions were convolved with a canonical hemodynamic response function to form regressors for the linear model. Parameter estimates (i.e. beta images) were assessed with least square regression analysis (e.g. Worsley and Friston, 1995). Our contrasts of interest were the main effect of semantic categorisation and perceptual matching conditions (relative to fixation). These contrast images represent summary images (i.e., contrast of maximum likelihood parameter estimates or the weighted beta images, see Poline, 2003) for the effects of interest. Because the fixation condition was not explicitly modelled as a regressor in our design matrix, the contrast images were equal to the beta images assessed by least square regression analysis. Thus, from this first-level analysis, these contrast (beta) images quantify how the fMRI signal changes with respect to the regressors and relative to an implicit baseline (here the baseline is coded in the constant term of our general linear model and includes all the unmodelled brain activity including the fixation). In other words, these contrast images are obtained by a cognitive subtraction between semantic words and an implicit baseline (fixation) but critically no other comparisons of conditions (i.e. subtractions) occurred in the second-level analysis during the fuzzy clustering.

### Second-level analyses of all conditions using cognitive subtractions

In the cognitive subtraction approach, two contrast images for each subject were entered in a second-level ANOVA with four conditions, two for the subjects who responded with their right hand and two for the subjects who responded with their left hand. We then computed the main effects of subject group, task and the main of effect of all conditions relative to fixation.

For the purposes of this paper, the results of this ANOVA serve only to provide *a priori* expectations for the clustering algorithm. Therefore, because our clustering algorithm used data collected during semantic decisions on written words only, our cognitive conjunction analysis focuses on identifying the functional networks required by this task. At a minimum these will include areas involved in low- and high-level visual processing, semantic associations, categorisation and a finger press response. Using the cognitive subtraction analysis, we identified these networks as follows:

• Finger press response areas were identified in the main effect of subject group which compared activation for the subjects who responded with (a) their left hand to the subjects who responded with their right hand and (b) vice versa.
• Visuo-attentional and perceptual processing areas were identified as those that were activated by (a) all conditions relative to fixation, after excluding all voxels that were differentially activated by type of task or subject group; and (b) the main effect of perceptual matching relative to semantic decision.
• Semantic associations were identified by the main effect of task which compared semantic to perceptual decisions.
• Deactivations (relative to fixation) were identified that were either common to all conditions or specific to semantic categorisations.
• Finally, we assessed the effect of age on semantic categorisation by including the age of the participants as a covariate in an SPM regression analysis.

Significant results from the cognitive subtractions are reported at *p* < 0.05 with a correction for multiple comparisons across the whole brain made on the basis of extent (minimum cluster size of 65 voxels at *p* < 0.001 uncorrected).

### Second-level clustering

For voxel selection, we ran a one sample *t*-test (i.e. random-effects analysis) on the contrast images for semantic decisions on words. Data for all subjects were pooled irrespective of whether they responded with their left or right hand. All other conditions (i.e. perceptual conditions) were excluded to avoid mixing between- and within-subject variability. From our between-subject second-level analysis, we generated a statistical parametric map of the *F* statistic at each voxel SPM{*F*}, which characterised differences in activation for semantic decisions on words relative to fixation over the whole group. Our clustering analysis was limited to those voxels with an *F* value > 5 (see section 1 of the Supplementary material for more details). No other conditions were entered into the clustering algorithm. Note that the beta values for semantic decision on words have negligible dependency on other conditions in the first-level analysis (e.g. perceptual matching) because the correlations between regressors were very weak in our block design. Thus, the parameter estimations of the beta values, with least square regression analysis, scale the contribution of each regressor to the data and therefore cannot be explained by a mixture of other regressors.

The clustering algorithm was based on the popular Fuzzy C-mean (FCM) clustering approach (Bezdek, 1981; Bezdek et al., 1997). See section 2 of the Supplementary material for more details. A detailed description of FCM algorithm can be found elsewhere (e.g. Bezdek et al., 1997; Fadili et al., 2001; Golay et al., 1998). The parameter “m” controlling the degree of fuzziness was set to 1.5 throughout this study (Fadili et al., 2001). For appropriate clustering of the contrast images (i.e. second-level clustering), we used the hyperbolic correlation of Golay et al. (1998) as a measure of similarity between voxels. It is interesting to note that this measure of similarity is based on the computation of the Pearson correlation coefficient between the BSVP of each voxel and the centroid of each cluster, irrespective of the mean (i.e. group) effect in that voxel.

Before running the FCM algorithm, we first ensured that each subject had normal activation levels in order to avoid some clusters being dominated by outlier subjects. This was achieved by using a modified fuzzy clustering approach that allows the detection of outlier subjects (Seghier et al., 2007). From this analysis, we excluded two outlier subjects which left 37 subjects for second-level clustering (for more details about this procedure, see section 3 of the Supplementary material). Critically, the “true” number of clusters (i.e. optimal number of classes) is unknown in FCM. To determine the most likely number of clusters, we used a previously described cluster-validity index that derives the optimal number of clusters in an unsupervised manner (Rezaee et al., 1998; Sun et al., 2004). Practically, FCM was repeated several times with the number of clusters varying from 2 to 39, the optimal number of clusters for our dataset was that when the cluster validity index was at a minimum. For more details see section 4 of the Supplementary material.

### Motivation for using the fuzzy clustering approach

To dissociate functional networks on the basis of between-subject variability, we needed a method that was data-driven, unsupervised, and based on an appropriate similarity metric (here the correlation between BSVPs). We chose the popular FCM algorithm (as detailed in section 2 of the Supplementary material) because it is (i) easy to implement for diverse measures of similarity (here the hyperbolic correlation distance), (ii) robust when the number of expected clusters is high, and (iii) ensures rich and fine segregation of different subcomponents (high sensitivity). These issues are detailed in some previous fMRI studies that compared FCM to ICA (e.g. Dimitriadou et al., 2004; Meyer-Baese et al., 2004; Smolders et al., 2007). Furthermore, we ensured an optimal implementation of the FCM algorithm by (i) performing an unsupervised search for the optimal number of clusters (as detailed in section 4 of the Supplementary material), (ii) setting the parameter “m” controlling the degree of fuzziness to a value within the range of values commonly used in FCM on fMRI datasets (e.g. Fadili et al., 2000, 2001; Golay et al., 1998; Moller et al., 2002), (iii) using a highly sensitive similarity metric to identify correlated BSVPs (see Fig. 7 in Golay et al., 1998), (iv) increasing the sensitivity of the clustering by focusing mainly on meaningful voxels (see section 1 of the Supplementary material and for a similar rationale see Fadili et al., 2000; Goutte et al., 1999; Gu et al., 2005; Moller et al., 2002; Seghier et al., 2007), (v) ensuring a high reliability of the clustering by removing outliers (i.e. deviant observations that may drive the calculation of the centroids) (e.g. for a discussion see Dave and Krishnapuram, 1997; Kharin, 1997; Leski, 2003; Salem and Nandi, in press).

## Results

### Task behaviour

Task accuracy and mean response times for semantic decisions on written words were 92 ± 6% and 1737 ms (range = 1264–2455 ms).

### Cognitive subtractions on activation and baseline conditions

**Fig. 3:**
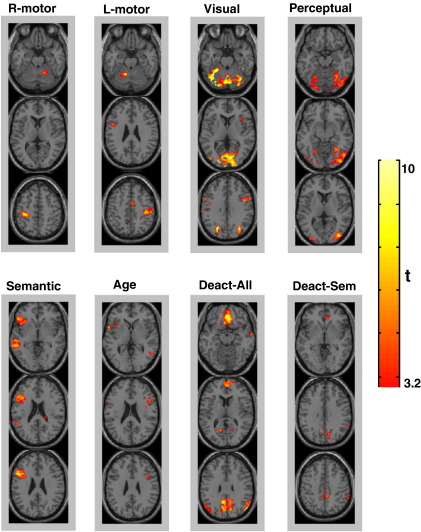
Cognitive subtractions results: for each pattern, identified regions are shown in red-to-yellow colour coding on three axial slices (at *p* < 0.001 uncorrected, minimum cortical volume of 0.52 ml (65 voxels)). See Results section for a list of these patterns.

To recap, these results serve only to provide *a priori* expectations for the clustering algorithm that was limited to semantic decisions on words only. In this context, we start by reporting the between-subject factor (i.e. the finger press response) because this allows us to explicitly test whether the second-level clustering approach (based on between-subject variance) can detect variability in our subjects that is known *a priori*. We then report increased or decreased activations for semantic categorisation relative to fixation or perceptual matching because these patterns will also be identified in the second-level clustering approach. A total of eight patterns obtained by cognitive subtractions are illustrated in Fig. 3.

#### Finger press response

As expected, sensorimotor cortex activation was contralateral to the hand used while cerebellar activation was ipsilateral to the hand used. Thus, subjects who used their right hand fingers increased activation in their left sensorimotor cortex and the right cerebellum (see “R-motor” in Fig. 3). In contrast, subjects who used their left hand fingers increased activation in the right sensorimotor cortex, SMA and the left cerebellum (see “L-motor” in Fig. 3).

#### Visual categorisation

Activation that was common to semantic and perceptual decisions relative to fixation, but not differentially activated by task or subject group, was identified in the cerebellum, medial occipital and superior parietal lobes (see “Visual” in Fig. 3). This pattern represented visuo-attentional and categorisation processes that were common to all conditions irrespective of group.

#### Perceptual processing

Activation for perceptual matching relative to semantic categorisations (i.e. unfamiliar symbols relative to familiar words) was observed in bilateral ventral–posterior middle occipital gyri with more extent in the right hemisphere (see “Perceptual” in Fig. 3). This pattern includes high-visual areas that are more involved in processing and matching unfamiliar visual stimuli.

#### Semantic associations

Activation for semantic relative to perceptual categorisations was observed in the left inferior frontal, left prefrontal and left posterior temporal lobe (see “Semantic” in Fig. 3). The corresponding but less significant effects in the homologue areas in the right hemisphere did not survive our statistical threshold. On the other hand, we also observed a significant positive relationship between age and semantic associations in mainly the right prefrontal, right precentral, right posterior temporal, left inferior frontal and cerebellum regions (see “Age” in Fig. 3).

#### Deactivation relative to fixation

Deactivation for semantic and perceptual conditions relative to fixation was observed in bilateral angular gyri, anterior and posterior cingulate cortex, medial frontal gyrus, precuneus, and the right anterior middle temporal gyrus, see “Deact-All” in Fig. 3. Deactivation (relative to fixation) for semantic decisions more than perceptual matching was observed in the posterior cingulate, right parietal lobule and medial frontal gyrus (see “Deact-Sem” in Fig. 3).

### Clustering approach on semantic categorisation of written words only

**Fig. 4:**
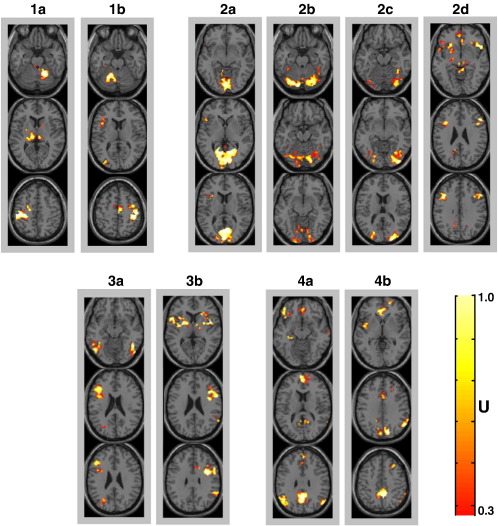
Second-level clustering results: for each cluster, from 1a to 5b, segregated networks (regions) are shown in red-to-yellow colour coding on three axial slices (only voxels with degree of membership *U* bigger than 1/3 are shown). See Results section for a list of these clusters.

The unsupervised FCM on all voxels with an *F* > 5 found the optimal number of networks to be 10 (i.e. optimal value for our cluster validity index; see Figure S3 of the Supplementary material). Fig. 4 illustrates these 10 networks which aggregate voxels with similar between-subject variability profiles (BSVPs). Before describing the predicted and nonpredicted networks below, there are two points to note. First, although we have numbered the networks 1a to 4b, their numbering is arbitrary as one network is not valued as more likely than another. We have therefore chosen to report them in an order that follows the order of the patterns in the cognitive subtractions results (as in Fig. 3). Second, the similarity measure used to cluster the voxels with FCM is based on correlations in BSVPs at the voxel level and thus independently from their mean values. In other words, the clustering was “blind” to whether the voxel was activated or deactivated relative to fixation.

#### Two networks associated with the finger press response

As predicted, motor regions associated with the finger press response were grouped in two separate clusters (Fig. 4): cluster 1a shows left sensorimotor cortex and the right cerebellum, and cluster 1b shows the right sensorimotor cortex, SMA and left cerebellum. The precise identification of these networks validates the clustering algorithm's ability to identify variance that was introduced *a priori* by including two different groups of subjects. These clusters are almost identical to the motor patterns obtained by cognitive subtractions. In addition, we note that cluster 1a also included unpredicted but plausible motor related activation in the thalamus. This may have been lost in the cognitive subtraction approach due to high within- or between-subject variability.

#### Four networks associated with visual categorisation

Cluster 2a included voxels from the medial occipital cortex in the vicinity of low-level visual areas (primary visual cortex and other secondary visual areas, e.g. V2). These regions are likely to be involved during the first step of visual processing of presented stimuli (i.e. written words). Remarkably, the second-level clustering was more successful at isolating these low-level (V1/V2) areas than cognitive subtractions where these areas were grouped with other high-level visual and attentional regions (e.g. see pattern “Visual” of Fig. 3). Moreover, these high-level visual areas were further segregated in other clusters. Ventral visual categorisation areas were identified in cluster 2b, including mainly voxels in the cerebellum, located bilaterally in lobule VI (Schmahmann et al., 2000), and the inferior occipital gyrus (Fig. 4). These regions are comparable to cerebellar regions that were implicated in visual categorisation irrespective of task or group (see pattern “Visual” of Fig. 3). Likewise, cluster 2c showed high-level visual areas located more laterally in the occipital lobe with more extent in the right hemisphere. These regions of cluster 2c were almost identical to the pattern “Perceptual” (Fig. 3) identified by cognitive subtractions of unfamiliar symbols relative to familiar written words. Finally, cluster 2d included bilateral insular, subcortical and frontal eye-field regions. This cluster may be involved in occulo-motor processing during the implicit reading of the three words presented as a triad. Note that the identified frontal eye-field regions overlap with the dorsal frontal regions identified by cognitive subtraction of effects that are common to all tasks (see top slice of pattern “Visual” in Fig. 3).

#### Two networks associated with semantic associations

The clustering algorithm identified one network that corresponded to areas with increased activation for semantic relative to perceptual categorisation (Fig. 4). Cluster 3a included the left prefrontal, left parietal and bilateral occipito-temporal cortex. This left dominant semantic network is comparable to the pattern identified by cognitive subtractions. The notable differences consisted of the detection of more foci in right occipito-temporal and left parietal lobule regions with clustering than cognitive subtractions, whereas the reverse was observed in ventral inferior frontal and posterior middle temporal regions (see cluster 3a of Fig. 4 and pattern “Semantic” of Fig. 3). Likewise, cluster 3b included right prefrontal cortex, right inferior parietal lobule, left inferior frontal gyrus, cerebellum and bilateral subcortical regions (Fig. 4). The BSVP of this cluster showed a very strong positive correlation with age (*r* = 0.65, *p* = 0.00001). This suggests that cluster 3b regions are more activated by older subjects during semantic association, and this corresponds to the age effects observed with cognitive subtractions (e.g. pattern “Age” of Fig. 3). The identification of cluster 3b is not unexpected as age (range 13–74 years) was deliberately introduced as an additional source of variability.

#### Deactivated networks

Cluster 4a consisted of regions that were mainly deactivated during the semantic decision task, including regions in the orbito-frontal or the medial frontal cortex, anterior and posterior cingulate cortex, bilateral angular gyrus, and the left ventral inferior frontal gyrus. These regions corresponded closely to those that were deactivated in the cognitive subtraction pattern “Deact-All” (Fig. 3). In contrast, cluster 4b (Fig. 4) included regions in the medial frontal, precuneus, posterior cingulate, left insula and right parietal lobule. These correspond to those showing stronger deactivation during semantic categorisation than perceptual matching tasks in the cognitive subtraction approach (pattern “Deact-Sem” of Fig. 3). The clustering, however, identified more regions in cluster 4b than cognitive subtractions (e.g. precuneus and left insula).

## Discussion

In this study, we present an alternative way of dissociating functional brain networks involved in the execution of a given cognitive task. Our rationale is based on the objective assumption that random subject to subject variations in activation patterns contain meaningful information about the level of involvement of different networks (e.g. Kherif et al., 2003; Miller and Van Horn, 2007; Nadeau et al., 1998; Seghier et al., 2007, 2008a). We demonstrate the feasibility of this approach using data from a relatively large number (i.e. 39) of subjects performing a popular semantic decision task. By looking at between-subject variability in brain activation, we successfully dissociated several networks that were differentially engaged during semantic decisions on written words without any comparison to other tasks or conditions. This contrasts to the well-known cognitive subtractions method that dissociates functional networks for a single task by comparing activation to experimentally designed baseline conditions (for more details about the problematic issue of an activation baseline, see Binder et al., 1999; Brandt, 2006; Newman et al., 2001; Stark and Squire, 2001). Decoding the between-subject variability with second-level clustering is a more “specific” way of characterising networks involved in the task of interest because it doesn't depend on how subjects are performing in other irrelevant tasks or conditions. Below, we discuss the meaningfulness of the networks segregated for the semantic task by directly comparing the results of second-level clustering to those identified using cognitive subtractions.

Before comparing second-level clustering to cognitive subtractions, it is important to keep in mind the fundamental differences between the two approaches (e.g. when comparing Fig. 3 to Fig. 4). First, as mentioned above, the second-level clustering findings are specific to the semantic decision on words, whereas the cognitive subtractions depended on the comparison of different conditions in our experimental design. Second, the computational framework in both techniques is fundamentally different because voxels are clustered on the basis of the similarity between their BSVPs irrespective of their average activation values. This means that the critical information is in the similarity of the shape of the variance across-subjects in different regions and this is not affected by the standard deviation of the between-subject variance. Consequently, when between-subject or within-subject variance is high, second-level clustering is likely to have higher sensitivity than cognitive subtractions.

In the context of our semantic task, we expected the involvement of the following processes: (i) visual and perceptual processing of presented written words, (ii) semantic associations between the words of the presented triad, (iii) categorisation and decision making, and (iv) finger press response. Each of these processes may divide into other expected or unexpected functional subcomponents. We start by discussing networks that correspond to the finger-press response in the cognitive subtraction analysis because this factor was deliberately manipulated between subjects which allows us to explicitly test whether the second-level clustering approach (based on between-subject variance) can detect known between-subject variability.

### Finger press response

As predicted, motor regions associated with the finger press response were segregated in two separate clusters 1a and 1b (Fig. 4) for right and left hand responses respectively. These clusters are almost identical to the motor patterns obtained by cognitive subtractions (patterns “R-motor” and “L-motor” in Fig. 3). Note, however, the interesting identification of the thalamus for right hand responses with second-level clustering (cluster 1a) that was not visible with cognitive subtractions (pattern “R-motor”), although it fits well with the known circuitry of voluntary finger movement (e.g. Jancke et al., 2000). The absence of this effect in Cluster 1b (the left hand motor response) may reflect the small number of subjects who made a left hand response (*n* = 12) which would limit the sensitivity of the clustering to detect consistency in the inter-subject variability. Conversely, the absence of thalamic activation in the cognitive subtractions (right-handed responders versus left handed responders or vice versa) could be a consequence of high between-subject variability in the mean activation difference. Indeed, if we lowered the statistical threshold (*p* < 0.05 uncorrected), thalamic activation was detected for each group (peak at *Z* = 2.8 in the left thalamus for right > left handed responders; *Z* = 2.5 in the right thalamus for left > right handed responders). As mentioned above, the successful identification of these motor networks validates the clustering algorithm's ability to identify variance that was caused by different processes in the between-subject dimension (i.e. variance introduced here *a priori* by including two different groups of subjects).

### Visual networks

Four networks (Fig. 4) were segregated including a set of low-level visual regions involved in the early step of visual processing of presented words (cluster 2a) and high-level visual and perceptual areas involved in global word processing and categorisation (clusters 2b and 2c). These areas are concordant with the well-known organisation of the visual system (e.g. Tootell et al., 1998). Interestingly, despite including one condition only, the second-level clustering was more successful at isolating this low-level visual activity (V1/V2 areas in cluster 2a) than the cognitive subtractions method (i.e. low-level areas were grouped with other visual areas in cognitive subtraction pattern “Visual” of Fig. 3).

It is interesting to note that the clustering of occipital voxels into low-level and high-level areas is concordant with previous work that identified comparable networks during rest after fMRI data decomposition across space, time and subjects (e.g. Calhoun et al., 2008; Damoiseaux et al., 2006; Salvador et al., 2005). The identified cerebellar regions in cluster 2b (Fig. 4) were also comparable to cerebellar regions in cognitive subtraction pattern “Visual” (Fig. 3), suggesting their potential role in visual categorisation processing (Calhoun et al., 2001). Likewise, regions in cluster 2c are almost identical to those that showed higher activation for matching unfamiliar symbols relative to familiar words in the cognitive subtractions (pattern “Perceptual” of Fig. 3). These regions have previously been observed during tasks that engaged matching visual stimuli (e.g. Bundesen et al., 2002; Gerlach et al., 1999) and their activity has been shown to be modulated by task difficulty (Gerlach et al., 1999). This suggests that the differences in activation during the processing of written words (as demonstrated by the clustering) may reflect differences in task difficulty (or efficiency) between subjects. Indeed, we observed that the BSVP (i.e. centroid) of cluster 2c was highly correlated to the in-scanner response times (RTs) of the semantic task (*r* = − 0.52, *p* = 0.001), suggesting that the involvement of these regions increased with efficient word processing.

Finally, cluster 2d included regions that were involved in occulo-motor processing (e.g. Lynch and Tian, 2005). These regions were grouped in the cognitive subtraction with other visual and categorisation areas that were common to semantic and perceptual decisions (pattern “Visual” of Fig. 3). To segregate (isolate) these regions with cognitive subtraction, additional control conditions would have been needed. This observation provides further support for the sensitivity of second-level clustering in segregating all the known processes involved in our semantic decision task.

### Semantic network

The left dominant pattern identified with cognitive subtractions (pattern “Semantic” of Fig. 3), when comparing semantic categorisation versus perceptual matching, is consistent with many previous studies (e.g. Billingsley et al., 2001; Cousin et al., 2007; Poldrack et al., 1999; Pugh et al., 1996; Ricci et al., 1999; Seghier et al., 2004). With second-level clustering, the semantic network (i.e. cluster 3a of Fig. 4) is comparable to this pattern, except that left middle temporal activation was weaker and ventral inferior frontal activation was absent. The apparent inconsistency was easily explained, however, because the posterior part of the ventral inferior frontal gyrus was clustered with the areas showing an effect of age (i.e. driven by older subjects in cluster 3b). In contrast, the anterior part was associated with the deactivated network (cluster 4a in Fig. 4). This is interesting because re-examination of the pattern “Semantic” (Fig. 3) in the cognitive subtraction analysis indicated that the strong semantic effect in this anterior part was in fact partly driven by deactivation in the perceptual matching conditions. In summary, these differences between the two methods could easily be understood in view of the fundamental differences between a method with an *a priori* knowledge and a data-driven method (e.g. see illustrations in Calhoun et al., 2001; McKeown et al., 1998).

A second cluster (3b) also identified parts of the known semantic network that was primarily activated by older subjects in the semantic decision task (e.g. Aizenstein et al., 2006; Marsh et al., 2006). Critically, the identification of cluster 3b highlights the sensitivity of the between-subject dimension to tease out the effect of demographic variables (e.g. age).

### Deactivated networks

Remarkably, although the clustering was “blind” to whether the voxel was activated or not relative to fixation, deactivated regions were clustered together into two separate clusters (clusters 4a and 4b, Fig. 4). These deactivated regions, including the medial frontal cortex, anterior and posterior cingulate cortex, and bilateral angular gyri, were comparable to the “default” network that might be “suspended” when subjects are engaged in a task (e.g. Damoiseaux et al., 2006; Fransson, 2006; Greicius et al., 2003; Raichle et al., 2001; Salvador et al., 2005; van den Heuvel et al., 2008). Interestingly, the segregation of deactivated regions in two different networks (i.e. clusters 4a and 4b) is in line with recent fMRI studies that segregated different parts of the anterior and posterior cingulate cortex and the medial frontal cortex into separate clusters (Calhoun et al., 2008; van den Heuvel et al., 2008). Moreover, Calhoun et al. (2008) showed that these two resting state networks were also consistently identified when subjects performed an auditory oddball task. In our case, the notable difference between clusters 4a and 4b was that cluster 4a included regions deactivated irrespective of task or group (similar to cognitive subtraction pattern “Deact-All” of Fig. 3), whereas cluster 4b included regions that were more deactivated during the semantic than perceptual matching task (i.e. regions identified in pattern “Deact-Sem” of Fig. 3). In sum, the dissociation of deactivated regions, as observed with cognitive subtractions, was mirrored in the second-level clustering.

### Potential applications

We have shown here how clustering fMRI data in the between-subject dimension can complement the widely-used cognitive subtraction approach. The advantage of second-level clustering is that it can identify networks that were not explicitly modelled by appropriate control conditions in the experimental design (e.g. clusters 2a, 2b, and 2d). It can also detect areas where the significance of the effect is low in the cognitive subtraction approach because of high within- or between-subject variance. In addition, our second-level clustering approach provides a very useful means to study clinical populations who are unable to switch between multiple tasks or uncomfortable staying in the scanner for long periods of time (e.g. by limiting the acquisition time to the main conditions of interest). Moreover, by studying the networks identified in different populations, abnormalities can be investigated at the systems rather than the regional level (e.g. by searching for similarities and differences between the identified clusters/networks in different populations). Our second-level clustering approach may therefore provide systems level signatures for different clinical populations.

Another clinical application would be to use the second-level clustering to identify different subtypes of patients within the same population. A broad literature has already shown that the same task can be implemented by different cognitive strategies in both diseased and healthy populations (e.g. Berl et al., 2005; Bluhm et al., 2007; Calhoun et al., 2008; Friston and Price, 2003; Kherif et al., in press; Noppeney et al., 2004; Price and Friston, 2002; Rombouts et al., 2005; Waites et al., 2006; Wang et al., 2007). These cognitive differences will have repercussions on the between-subject variance which might enable our second-level clustering approach to dissociate patient subtypes without the need for multiple task and baseline measurements. For instance, it may be the case that the centroids of some specific networks are driven by a subgroup of patients, which would suggest that specific networks have been engaged by this subgroup. This was illustrated in our own data where we found right hemisphere networks that were more activated in older than younger subjects. Thus, by dissociating subjects in terms of their activation pattern, we were able to examine post-hoc how these subjects differed on other measures (e.g. behavioural or demographic).

In the same way, the second-level clustering approach should help to dissociate and characterise the possible degenerate systems that support the same task (i.e. many-to-one structure-to-function relationships, see for more details Friston and Price, 2003; Noppeney et al., 2004; Price and Friston, 2002). This would be a valuable contribution to patient studies that are generally interested in how to predict or characterise the different mechanisms of recovery. For instance, by identifying the different networks (i.e. degenerate systems) that sustain the execution of a given function, one could predict that damage to a particular region may cause abnormally low activity in other intact regions of the same network (e.g. the disconnected network) and/or abnormally high activity in a set of regions of the remaining networks (e.g. compensatory networks) (see examples in Price et al., 2003, 2001).

### Practical issues

Some practical issues should be acknowledged when performing second-level clustering. Specifically, this approach based on only one representative value per subject: (i) ignores possible fluctuations of networks over time, for instance when subject's strategies vary between trials (e.g. Fox et al., 2007). This however should not be a problem if there are sufficient trials with successful performance; (ii) considers that anatomical inter-individual variability is negligible after spatial normalisation and smoothing. This assumption is reasonable as the scale of the anatomical variability is much smaller (e.g. Juch et al., 2005; Zilles et al., 1997) compared to the large-scale networks that we observed here in the between-subject dimension; (iii) is sensitive to the presence of outlier subjects who might dominate the segregation of networks (e.g. Seghier et al., 2007; Wager et al., 2005), and for this reason we excluded outliers in our current sample before clustering; (iv) relies on the method used for clustering (ICA, fuzzy clustering) and the determination of the expected/optimal number of classes/components (e.g. Dimitriadou et al., 2004; Smolders et al., 2007).

Furthermore, one important issue concerns the assessment of the reliability/consistency of the clustering algorithm. Several approaches have previously been proposed (e.g. Breckenridge, 1989; Duann et al., 2003; Meinecke et al., 2002; Salem and Nandi, in press; Ylipaavalniemi and Vigário, 2008); in the context of second-level clustering, different practical procedures might be very useful. First, removing outlier subjects (see Methods section above for more details) will help to guarantee a good reliability of the clustering. Second, depending on initial conditions (Bezdek, 1981), the FCM algorithm may converge to different fuzzy partitions (i.e. local minima, see illustration in Fig. 1 of Ylipaavalniemi and Vigário, 2008) and may lead to the detection of spurious partitions (Moller et al., 2002). One possible way to overcome this problem is to iterate the FCM algorithm on the same dataset with several different random initialisations (for more details see Chuang et al., 1999; Moller et al., 2002; Pena et al., 1999) as it is unlikely that different initialisations will lead to the same local minima. Thus, randomising the initial conditions allows the optimum solution to converge from different directions of optimisation. Third, using resampling methods, including bootstrap and delete-k Jackknife (Davidson and Hinkley, 1997; Efron and Tibshirani, 1993; Shao and Tu, 1995; Wu, 1986), would help to objectively assess the reliability/consistency of the identified clusters. For instance, it is feasible to check if the obtained clusters were consistent (or replicable) by repeating the clustering several times on all but (randomly) k subjects (for a similar rationale see Seghier et al., 2008a). However, when using these resampling methods, either by deleting k subjects or by sampling with replacement, it is critical to ensure that the generated subsamples are representative of the main features of the original dataset (i.e. here in terms of age, gender and motor output). For example, in our dataset, with a limited number of subjects who responded with their left hand (12 subjects), the motor output factor might not be sufficiently appreciated by the clustering of each generated subsample.

On the other hand, one main practical advantage of the clustering approach is its applicability for the existing fMRI studies with standard block or event-related paradigms. For example, it does not require particular acquisition schemes, as is the case for other data-driven approaches in the time domain where continuous rest or uncontrolled stimulation are usually employed (e.g. Bartels and Zeki, 2004; Damoiseaux et al., 2006; Fransson, 2006; Hampson et al., 2002; Hasson et al., 2004; Jaaskelainen et al., 2008; Malinen et al., 2007; van den Heuvel et al., 2008). The approach is also highly feasible in the context of the growing interest in databases that involve fMRI studies with large numbers of subjects (see Kiehl et al., 2005; Schapiro et al., 2004; Schmithorst et al., 2007; Szaflarski et al., 2006).

Finally, because no *a priori* knowledge was used in second-level clustering, the interpretation of identified clusters is more challenging than with cognitive subtractions (a common problem in data-driven approaches, see Chuang et al., 1999; Fadili et al., 2001; Gomez-Laberge et al., 2008). Here, we compared the identified clusters to the patterns found by cognitive subtractions, which was very helpful to assert a function/role to each cluster. However, having identified the function of the areas with one sample of participants scanned with multiple conditions, we can then scan a second population (e.g. patients) to establish how the normal networks are disrupted. In addition, demographic or behavioural variables can help explain/interpret the identified clusters. For instance, age effects were relevant for interpreting cluster 3b. In the same way, using in-scanner response times (RTs), we observed that the BSVP (i.e. centroid) of cluster 3a (the semantic network) was negatively correlated to the differences in RTs between the semantic and perceptual tasks (*r* = − 0.39, *p* = 0.02). We can therefore infer that the involvement of regions of cluster 3a increases with fast (efficient) semantic processing speed.

## Conclusion

We have shown here the feasibility and meaningfulness of using the between-subject dimension for segregating different functional networks for a semantic decision task. The identified networks included low- and high-level visual processing, semantic categorisation, decision making and motor response networks. Compared to the commonly used cognitive subtractions, our method showed high sensitivity despite only using one experimental condition. Our method can be used to reveal “hidden” or unpredicted networks that might be difficult to identify using standard cognitive subtractions between manipulated tasks/stimuli. Moreover, in addition to the existing literature about the resting-state networks, this work points to the importance of taking into account the operation of different networks that interact in the between-subject dimension during the execution of a particular cognitive task.
