# Supplementary material for: Dissociating functional brain networks by decoding the between-subject variability
Source: Neuroimage. 2009 Apr 1;45(2):349–59. doi: 10.1016/j.neuroimage.2008.12.017 (PMC2652023; doi:10.1016/j.neuroimage.2008.12.017)
Supplement: Supplementary material [file mmc1.pdf]

Supplementary Material:

1- Voxel selection:

One important issue in clustering is the selection of the number of voxels (noted *n*). Previous work has suggested that selecting a limited number of voxels might be useful for (i) reducing the high dimensionality of the clustering and improving computational robustness and speed; and (ii) increasing the sensitivity of the clustering by focusing mainly on meaningful voxels (i.e. avoiding artifactual or outlier voxels). In our analysis, we limited the volume of interest for the unsupervised fuzzy clustering to all voxels with F-value > 5 (p uncorrected < 0.032, d.f. = [1, 36]) in a one sample t-test of on the effect of semantic categorisation relative to fixation. This identified voxels that are affected by these conditions irrespective of the direction of the effect. It resulted in a total of approximately *n* = 77000 voxels for the FCM. Figure S1 illustrates the distribution of the selected *n* voxels.

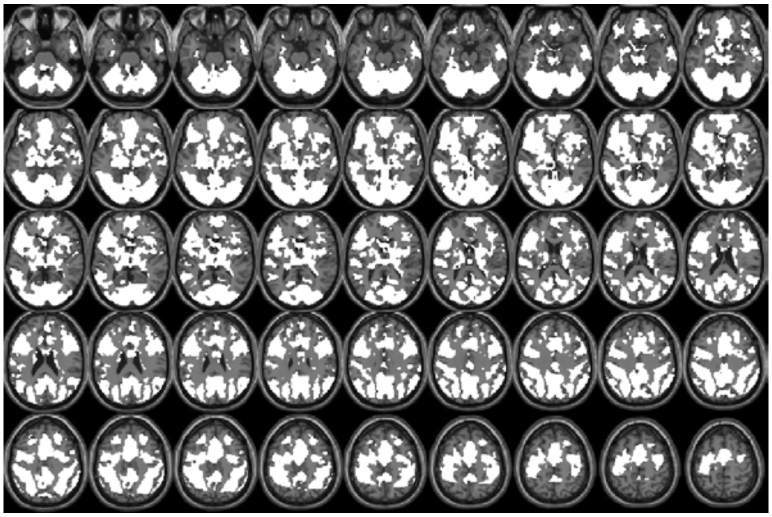

Mask (F > 5.0)

Figure S1. All voxels (in white) used in the second-level clustering projected on axial slices.

2- Fuzzy c-mean (FCM) algorithm:

In this work, we used the popular fuzzy c-mean (FCM) algorithm (Bezdek, 1981; Bezdek et al., 1997). In practice, we select *n* voxels that we want to assign to *c* clusters. Each voxel *i* has a vector X_i of *p* values that correspond to the number of properties (e.g. here, number of subjects). Each cluster *j* is characterised by a centroid V_j, which is its characteristic profile. The resemblance between each voxel *i* and each centroid V_j is assessed by the distance D_{ij} between X_i and V_j. The degree of membership U_{ij} is calculated for each voxel *i* by comparing D_{ij} for each cluster *j* to all other clusters.

Practically, the algorithm is based on minimising the following function J_m:

$$J_m = \sum_{i=1}^{n} \sum_{j=1}^{c} U_{ij}^m \cdot D_{ij}^2$$

where "*m*" is the degree of fuzziness.

Degree of membership *U* and centroids *V* are thus defined as:

$$U_{ij} = \frac{1}{\sum_{k=1}^{c} \left(\frac{D_{ij}}{D_{ik}} \right)^{2/m-1}}$$

$$V_j = \frac{\sum_{i=1}^{n} U_{ij}^m \cdot X_i}{\sum_{i=1}^{n} U_{ij}^m}$$

For appropriate clustering, the choice of distance (similarity) measure *D*, the degree of fuzziness *m* and the number of expected clusters are critical. In our context, we used the hyperbolic correlation distance proposed previously by Golay et al. (1998) in the context of first-level data-driven fMRI analysis. Accordingly, *D* is defined as (Fadili et al., 2000; Golay et al., 1998):

$$D_{ij} = \frac{1 - CC_{ij}}{1 + CC_{ij}}$$

Where CC_{ij} is the Pearson correlation coefficient between X_i and V_j.

3- Outlier subjects:

Before running the FCM algorithm on all subjects, we first checked that all subjects have normal activation levels in order to avoid some clusters being dominated by outlier subjects. For this aim, we used a modified fuzzy clustering approach that allows the detection of outlier subjects (for more details about this procedure, see (Seghier et al., 2007)). Practically, this procedure used fuzzy clustering to identify voxels that are dominated by only one subject (i.e. a given subject is an outlier at a given voxel). Then, a global measure, noted G, is estimated by computing a whole brain score that indicates how each subject is dominating in a relative sense the group activation pattern. Using a tuning factor equal to 3 ($\alpha = 3$ in equation (1) of Seghier al. 2007), Figure S2A illustrates the global measure G for our 39 subjects. Two subjects (number 16 and 39 in Figure S2A) had high G values. When these two subjects were included in the clustering of all voxels, the centroids of some clusters were clearly dominated by these subjects (see Figure S2B for an illustration). These centroids are not meaningful in the context of our second-level clustering because they represent the particular case of the activated pattern that is dominated by one subject. On this basis, we excluded these two outlier subjects which left 37 subjects for second-level clustering.

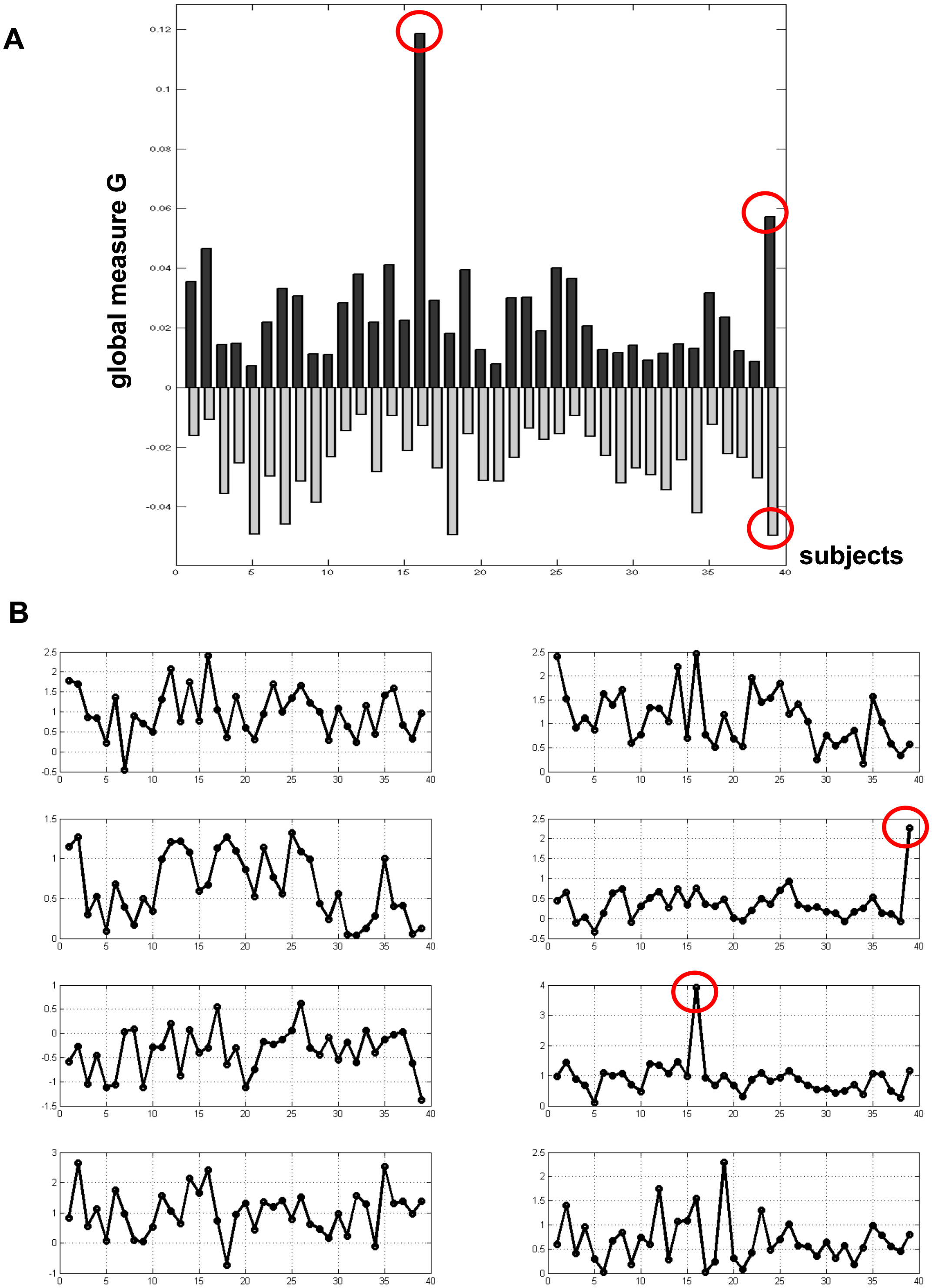

Figure S2. (A) the global measure G for all subjects. Outlier subjects are marked with a red circle. (B) centroids of 8 clusters when all subjects are included during the clustering with FCM. Subjects 16 and 39 clearly dominated two centroids (marked by a red circle).

4- Optimal number of clusters:

Critically, the "true" number of clusters (i.e. optimal number of classes) is usually unknown in FCM. In this perspective, several cluster-validity indices have previously been proposed in the literature to appreciate, in an unsupervised manner, the optimal number of clusters (for a review see (Wang and Zhang, 2007)). These indices combined different measures of compactness and separation of the clustering in order to ensure that identified clusters are compact and well-separated. In our context, we used a modified version of the Rezaee-Lelieveldt-Reider (RLR) cluster-validity index (Rezaee et al., 1998) as suggested previously by Sun et al. (Sun et al., 2004).

The Rezaee-Lelieveldt-Reider index (RLR) was defined as:

$$RLR = \frac{\sum_{j=1}^{c} \sigma_j}{c \cdot \|\sigma_X\|} + \frac{1}{\alpha} \cdot \left(\frac{V_{\max} \cdot SS}{V_{\min}} \right),$$

Where:

$$\sigma_j = \sum_{i=1}^{n} U_{ij} \cdot D_{ij}^2 \qquad SS = \sum_{j=1}^{c} \frac{1}{\sum_{k=1}^{c} \|V_j - V_k\|}$$

$$V_{\min} = \min_{j,k}\left(\|V_j - V_k\|\right) \qquad V_{\max} = \max_{j,k}\left(\|V_j - V_k\|\right)$$

The constant α is a weighting constant and σ_X is the variance of the whole data set. V_j, U_{ij} and D_{ij} are respectively the centroid of the j-th cluster, the degree of membership of the i-th voxel to the j-th cluster, and the distance between the i-th voxel and the j-th cluster. The best c-partition is obtained by minimising RLR with respect to the number of clusters *c*. In the original definition of RLR, the constant α was set to 1; however, here we set α equal to the value of $\frac{V_{d\max}}{V_{d\min}} \cdot SS$ when *c* reached the maximum number of clusters as suggested previously (for more details, see (Sun et al., 2004)).

Practically, FCM was repeated several times with the number of clusters varying from 2 to 39, and the number of clusters that minimise the RLR index were considered as

the optimal number of clusters for our dataset. Here, we found that the RLR cluster-validity index showed an optimal (minimum) value when the number of clusters was 10 (see Figure S3 below).

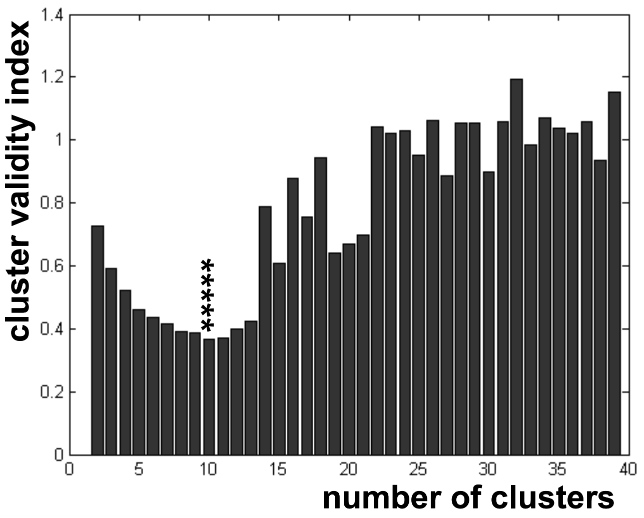

Figure S3. The optimal number of clusters (c=10, marked with stars) that minimised the RLR index.

References:

Bezdek, J.C., 1981. Pattern recognition with fuzzy objective functions algorithms. Plenum Press, New York.

Bezdek, J.C., Hall, L.O., Clark, M.C., Goldgof, D.B., Clarke, L.P., 1997. Medical image analysis with fuzzy models. Stat Methods Med Res 6, 191-214.

Fadili, M.J., Ruan, S., Bloyet, D., Mazoyer, B., 2000. A multistep unsupervised fuzzy clustering analysis of fMRI time series. Hum Brain Mapp 10, 160-178.

Golay, X., Kollias, S., Stoll, G., Meier, D., Valavanis, A., Boesiger, P., 1998. A new correlation-based fuzzy logic clustering algorithm for fMRI. Magn Reson Med 40, 249-260.

Rezaee, M.R., Lelieveldt, B.P.F., Reider, J.H.C., 1998. A new cluster validity index for the fuzzy c-mean. Pattern Recogn Lett 19, 237-246.

Seghier, M.L., Friston, K.J., Price, C.J., 2007. Detecting subject-specific activations using fuzzy clustering. Neuroimage 36, 594-605.

Sun, H., Wang, S., Jiang, Q., 2004. FCM-based model selection algorithms for determining the number of clusters. Pattern Recogn 37, 2027-2037.

Wang, W., Zhang, Y., 2007. On fuzzy cluster validity indices. Fuzzy Sets Sys 158, 2095-2117.
